# Estrogen/ERα signaling axis participates in osteoblast maturation via upregulating chromosomal and mitochondrial complex gene expressions

**DOI:** 10.18632/oncotarget.23453

**Published:** 2017-12-19

**Authors:** Pei-I Lin, Yu-Ting Tai, Wing P. Chan, Yi-Ling Lin, Mei-Hsiu Liao, Ruei-Ming Chen

**Affiliations:** ^1^ Graduate Institute of Medical Sciences, College of Medicine, Taipei Medical University, Taipei, Taiwan; ^2^ Cell Physiology and Molecular Image Research Center and Department of Anesthesiology, Wan Fang Hospital, Taipei Medical University, Taipei, Taiwan; ^3^ Department of Radiology, Wan Fang Hospital, Taipei Medical University, Taipei, Taiwan; ^4^ Anesthesiology and Health Policy Research Center, Taipei Medical University Hospital, Taipei, Taiwan

**Keywords:** estrogen/ERα, osteoblast mineralization, ATP synthesis, genomic complex genes, mitochondrial COX I

## Abstract

Estrogen deficiency usually leads to bone loss and osteoporosis in postmenopausal women. Osteoblasts play crucial roles in bone formation. However, osteoblast functions are influenced by mitochondrial bioenergetic conditions. In this study, we investigated the roles of the estrogen and estrogen receptor alpha (ERα) axis in mitochondrial energy metabolism and subsequent osteoblast mineralization. Exposure of rat calvarial osteoblasts to estradiol caused substantial improvements in alkaline phosphatase activities and cell calcification. In parallel, treatment of human osteoblast-like U2OS cells, derived from a female osteosarcoma patient, with estradiol specifically augmented ERα levels. Sequentially, estradiol stimulated translocation of ERα to nuclei in human osteoblasts and induced expressions of genomic respiratory chain complex *NDUFA10*, *UQCRC1*, cytochrome c oxidase (*COX*)*8A*, *COX6A2*, *COX8C*, *COX6C*, *COX6B2*, *COX412*, and *ATP12A* genes. Concurrently, estradiol stimulated translocation of ERα to mitochondria from the cytoplasm. A bioinformatic search found the existence of four estrogen response elements in the 5’-promoter region of the mitochondrial *cox i* gene. Interestingly, estradiol induced COX I mRNA and protein expressions in human osteoblasts or rat calvarial osteoblasts. Knocking-down ERα translation concurrently downregulated estradiol-induced COX I mRNA expression. Consequently, exposure to estradiol led to successive increases in the mitochondrial membrane potential, the mitochondrial enzyme activity, and cellular adenosine triphosphate levels. Taken together, this study showed the roles of the estradiol/ERα signaling axis in improving osteoblast maturation through upregulating the mitochondrial bioenergetic system due to induction of definite chromosomal and mitochondrial complex gene expressions. Our results provide novel insights elucidating the roles of the estrogen/ERα alliance in regulating bone formation.

## INTRODUCTION

Osteoporosis, one of the most impactful metabolic diseases in the elderly, is also called a silent disorder that is characterized by a decrease in the bone mineral density (BMD) and a T score of ≤ -2.5 [1]. According to statistical stratification of the International Osteoporosis Foundation, one-third of postmenopausal women may suffer from osteoporosis. In the clinic, vertebral fractures are a common complication and a lethal factor for osteoporotic patients [2]. Osteoporosis is attributed to multiple risk features, including genetics, lifestyle, the nutritional status, and personal and family histories. Among these factors, the aging-induced deficiency of estrogen, a major hormonal regulator of bone metabolism, plays a critical role in the pathophysiological incidence of osteoporosis [3]. Healthy bones are dynamically balanced by a process of bone remodeling involving osteoblast-mediated bone formation and osteoclast-mediated bone resorption [4]. An imbalance of bone remodeling may lead to bone diseases, such as osteoporosis and bone defects. Conventionally, a shortage of estrogen can stimulate osteoclastogenesis via activation of a receptor activator of the nuclear factor kappa-B ligand pathway [5, 6]. Moreover, estrogen can improve bone formation by increasing osteoblast lifespans [6, 7]. As a result, an estrogen deficiency in postmenopausal women induces bone resorption but represses bone formation. In osteogenesis and osteoclastogenesis, estrogen receptor alpha (ERα) functions as an effective receptor to transduce estrogen-induced messages in osteoblasts and osteoclasts [7]. Nakamura et al. reported that when depleting ERα, trabecular bone mass is instantaneously lost [8]. A recent study showed that aging can reduce ERα-directed mitochondrial suppression of glutamine anaplerosis and osteogenic differentiation in mesenchymal stem cells [9]. Accordingly, the estrogen/ERα signaling pathway is essential for maintaining bone health.

Adenosine triphosphate (ATP), an end product of aerobic respiration, functions as a coenzyme for intracellular energy transfer [10]. Mitochondria with double membranes provide an appropriate environment for ATP biosynthesis. A series of complexes, i.e., complexes I-IV, and ATP synthase are anchored in inner membranes of mitochondria to process respiratory electron chain reactions and consequent ATP production [10, 11]. These mitochondrial complexes are organized by multifaceted proteins encoded by chromosomal and mitochondria genes [12]. In human mitochondria, there are 44, 4, 11, and 14 structural units that contribute to construction of complexes I, II, III, and IV, respectively [10, 12]. Among these human mitochondrial complex subunits, 7, 4, and 14 proteins are encoded by mitochondria to separately build up complexes I, III, and IV. In particular, cytochrome c oxidase (COX) I is one of three core components that construct redox-active centers in complex IV with other nuclear-encoded proteins [11]. A variety of intrinsic and extrinsic factors contribute to regulating expressions of these genomic and mitochondrial DNA-encoding complex genes [13]. Estrogen, one of these factors, is involved in mitochondrial energy metabolism. For example, estrogen is thought to be a master regulator of bioenergetic systems in the brain that control glucose transport and glycolysis [14]. Furthermore, estrogen can improve mitochondrial activities and neural health, so administration of this hormone may prevent Alzheimer’s disease [15, 16]. Hsieh et al. reported that sex hormones will be a novel therapeutic adjunct for traumatic injury since they can increase mitochondrial bioenergetics [17]. Notably, cellular ATP production and systemic estrogen levels decline with aging, to < 10 pg/mL in postmenopausal women [18]. Nevertheless, studies of connections among estrogen deficiency, bone remodeling, and mitochondrial bioenergetics are limited.

ERα, a ligand-activated transcription factor, is the major regulator transducing the effects of estrogen on bone metabolism [19]. Structurally, ERα is composed of several functional domains that are crucial for hormone binding, DNA association, and activation of transcription [20]. Following binding with estrogen to the hormone domain, ERα is functionally activated and then translocated to nuclei or mitochondria [14, 21]. Concurrently, the ligand-activated ERα forms a dimer to induce multiple gene expressions by binding to estrogen response elements (EREs), a palindromic inverted repeat of 5′-GGTCAnnnTGACC-3′, existing in the 5’-promoter regions of these specific genes. In healthy bones, ERα participates in maintaining a dynamic balance of bone remodeling through raising nuclear-initiated induction of the death ligand Fas gene that results in osteoclast apoptosis [22]. Otherwise, ERα dysfunction because of an estrogen deficiency reduces levels of the Fas protein and induces osteoclastogenesis and consequent an osteoporotic pathophysiology. Our previous study also showed that estrogen-activated ERα can induce expressions of cell differentiation-related bone morphogenetic protein-6, collagen type I, and osteocalcin genes in primary rat osteoblasts and accordingly stimulate cell maturation [23]. Recently, Huang et al. reported that ERα can increase mitochondrial glutaminase expression in mesenchymal stem cells [9]. However, the effects of the estrogen and ERα axis on regulating expressions of human mitochondrial energy metabolism-related complex genes are still little known. Thus, this study was aimed to investigate participation of the estrogen/ERα signaling axis in osteoblast maturation and possible mechanisms, especially in regulating expressions of genomic and mitochondrial complex genes.

## RESULTS

### Estradiol improves ALP activity and osteoblast mineralization

**Figure 1:**
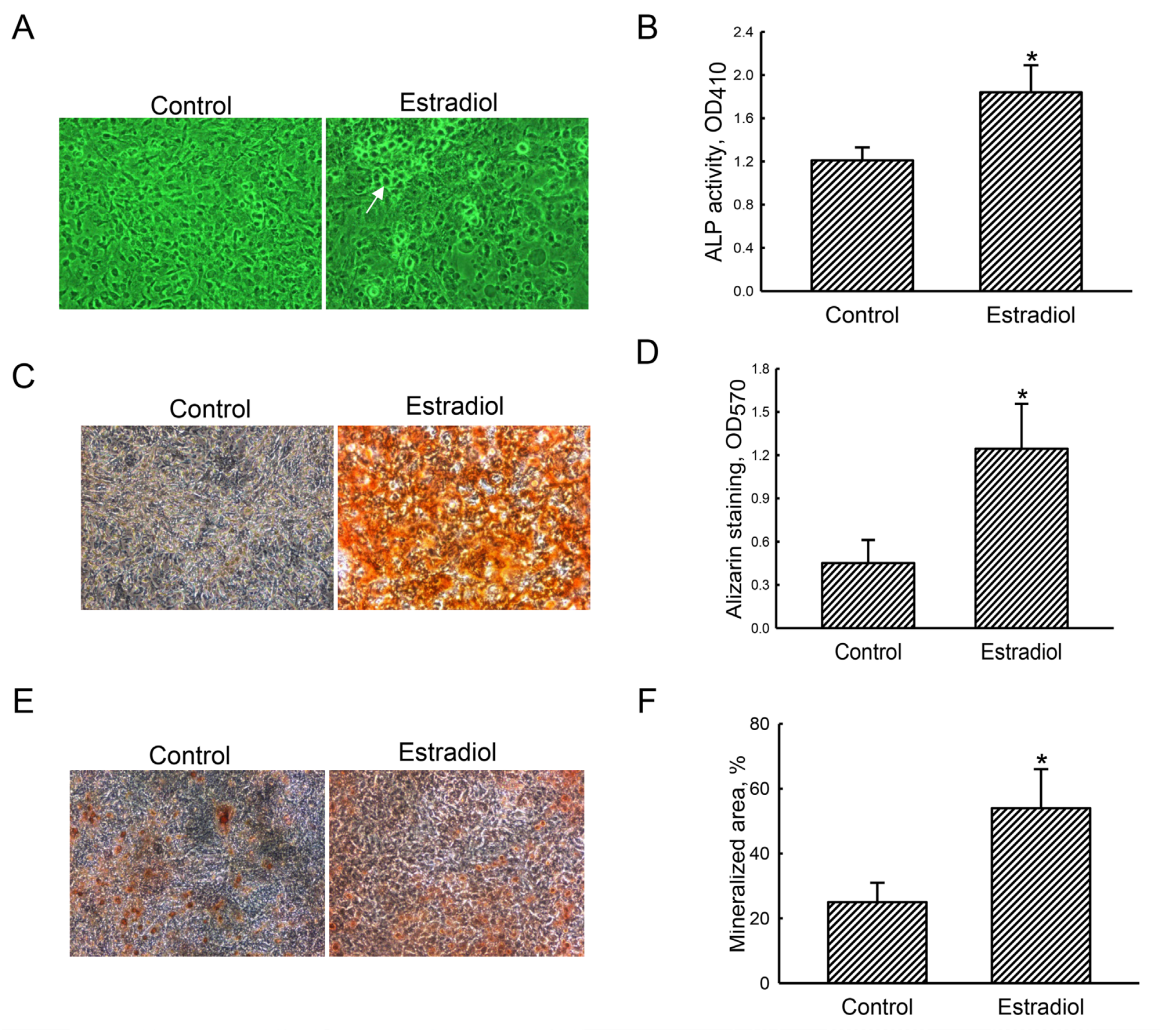
Effects of estradiol on osteoblast maturation Primary rat osteoblasts isolated from neonatal calvarias were exposed to a combination of estradiol (10 nM) and the differentiation agent, including dexamethasone, ascorbic acid, and β-glycerophosphate, for 21 days. Control cells received the differentiation agent only. Cell morphology was observed using a light microscope **(A)**. The symbol, →, indicates a calcified nodule. Alkaline phosphatase (ALP) activity was assayed with a colorimetric method **(B)**. Mineralized nodules were stained using Alizarin red S- **(C)** and the von Kossa-staining **(E)** protocols. These nodule signals were quantified and statistically analyzed **(D** and **F)**. Each value represent the mean ± SEM for *n* = 6. The symbol ^*^ indicates that the value significantly differed from the respective control group, *p* < 0.05. 100x.

After exposure to the differentiation agent for 21 days, primary rat osteoblasts had grown compactly (Figure 1A, left panel). In contrast, rat calvarial osteoblasts were co-treated with estradiol and the differentiation agent produced calcified nodules (right panel). Furthermore, treatment of primary osteoblasts with estradiol led to a significant 55% increase in ALP activity compared to the control group (Figure 1B). Results of Alizarin red S- and von Kossa-staining protocols revealed that exposure of primary rat osteoblasts to estradiol induced greater cell mineralization (Figure 1C, 1E, right panels). These signals were quantified and statistically analyzed (Figure 1D, 1F). Administration of estradiol improved osteoblast calcification by 2.8- and 2.2-fold in the Alizarin red S- and von Kossa-staining protocols, respectively.

### Estradiol specifically augmented levels of cellular ERα in human osteoblasts

**Figure 2:**
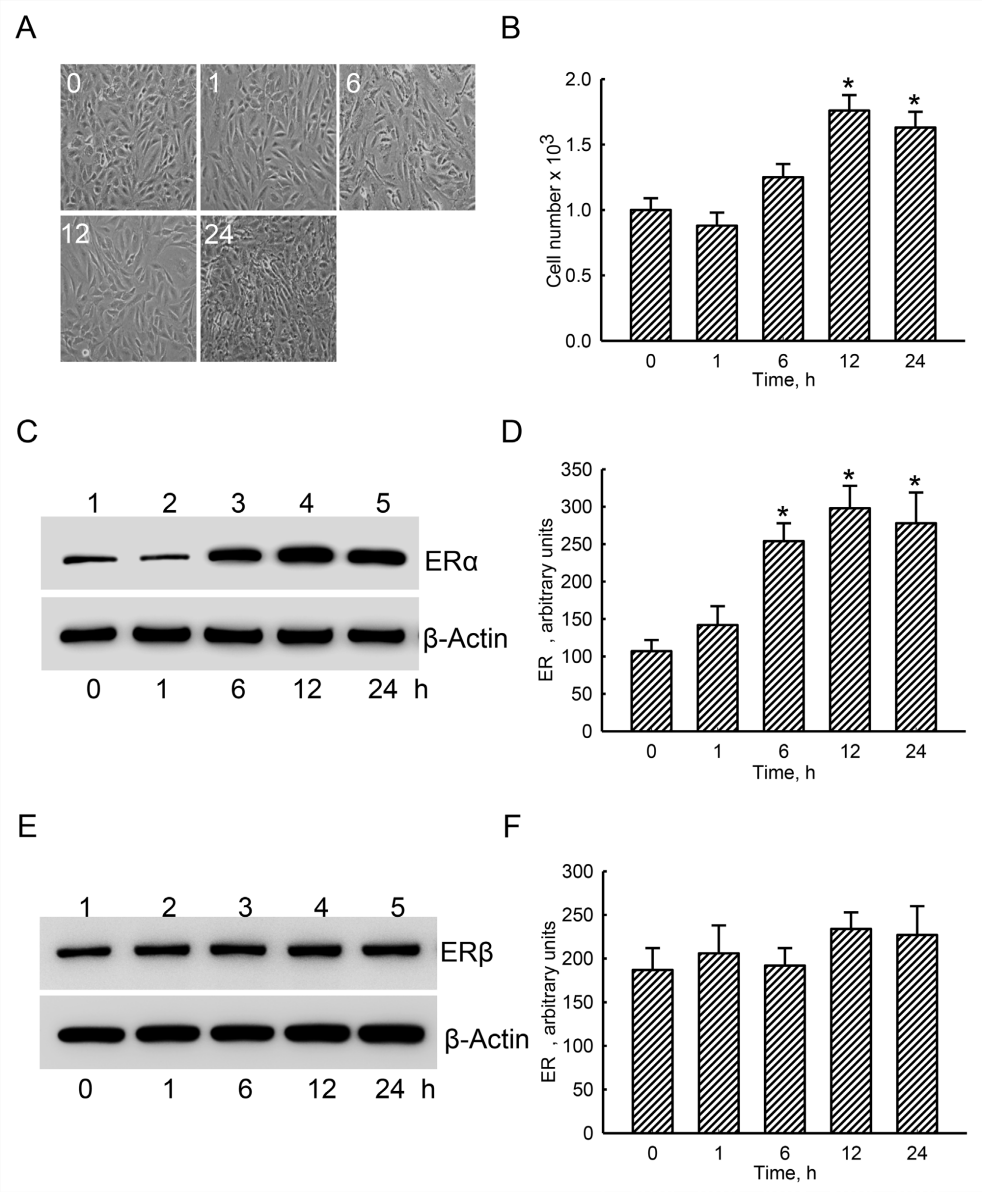
Effects of estradiol on levels of estrogen receptor alpha (ERα) and ERβ proteins Human osteoblast-like U2OS cells were exposed to 10 nM of estradiol for 1, 6, 12, and 24 h. Cell morphology was observed with a light microscope **(A)**. Cell proliferation was analyzed using a trypan blue exclusion assay **(B)**. After drug administration, cellular proteins were prepared for immunoblot analyses. Levels of ERα and ERβ were immunodetected **(C** and **E, top panels)**. Amounts of β-actin were analyzed as the internal standard **(C** and **E, bottom panels)**. These protein bands were quantified and statistically analyzed **(D** and **F)**. Each value represents the mean ± SEM for *n* = 6. The symbol ^*^ indicates that the values significantly (*p* < 0.05) differed from the respective control group, *p* < 0.05.

Exposure of human osteoblast-like U2OS cells to estradiol for 1 and 6 h did not affect the cell morphology or cell number (Figure 2A, 2B). In contrast, after treatment with estradiol for 12 and 24 h, numbers of human osteoblasts were augmented by 76% and 69%, respectively (Figure 2A, 2B). ERα was detected in the untreated group (Figure 2C, top panel, lane 1). Administration of estradiol to human osteoblasts for 1 h did not change ERα levels (lane 2). However, after exposure for 6, 12, and 24 h, levels of ERα in human osteoblasts obviously increased (lanes 3-5). β-Actin was immunodetected as the internal standard (bottom panel). These protein bands were quantified and statistically analyzed (Figure 2D). Treatment of human osteoblasts with estradiol for 6, 12, and 24 h led to significant 2.4-, 2.8-, and 2.6-fold increases in cellular ERα levels, respectively. In comparison, ERβ was also immunodetected in untreated human osteoblasts (Figure 2E, top panel, lane 1). However, amounts of ERβ were not influenced by estradiol administration (lanes 2-5). Statistical analyses of these protein bands using β-actin as the internal control showed that estradiol did not affect levels of ERβ in human osteoblasts (Figure 2F).

### Estradiol stimulated translocation of ERα from the cytoplasm to nuclei

**Figure 3:**
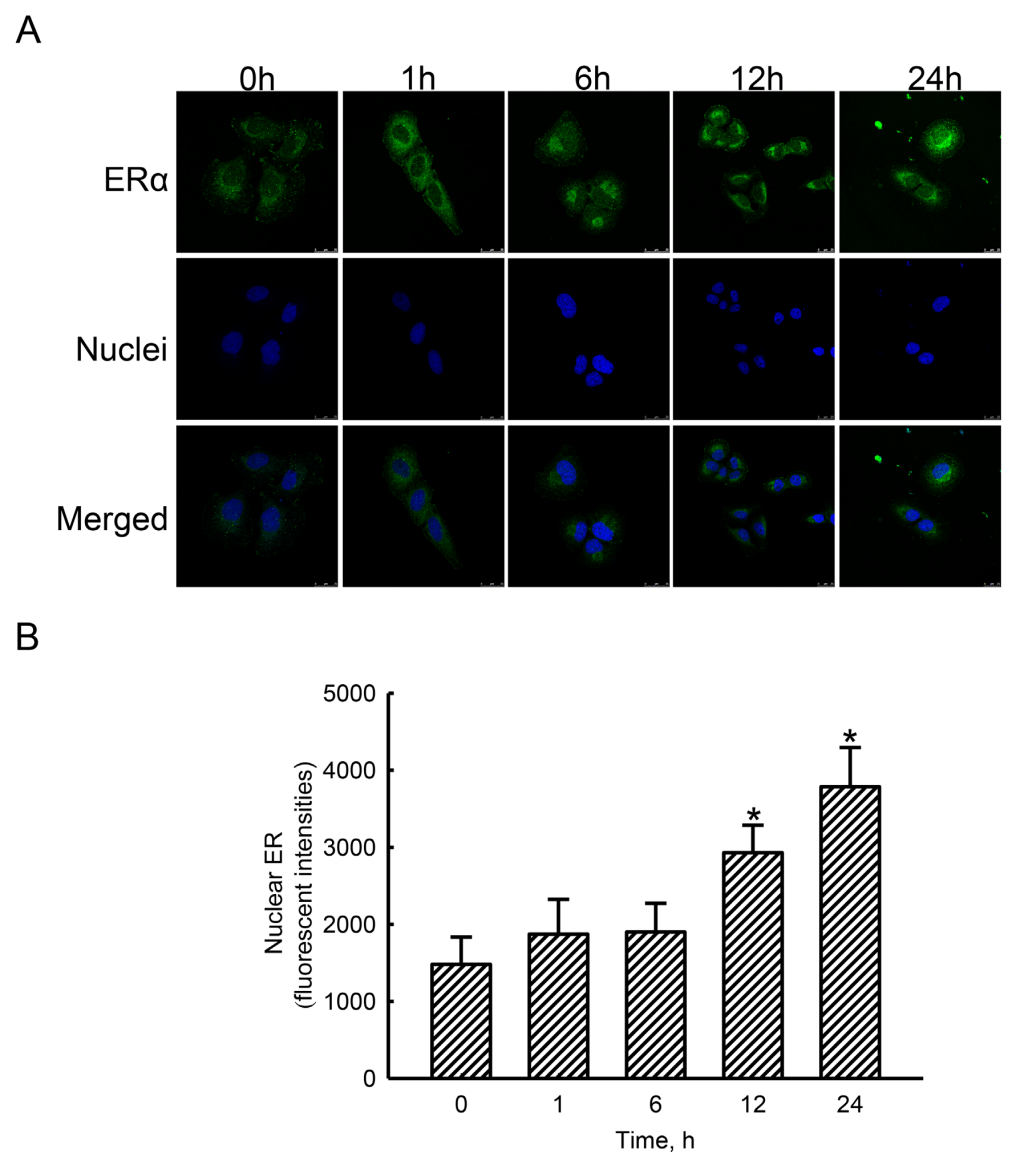
Effects of estradiol on translocation of estrogen receptor alpha (ERα) to nuclei Human osteoblast-like U2OS cells were exposed to 10 nM of estradiol for 1, 6, 12, and 24 h. Distribution of the ERα protein in human osteoblasts was immunodetected using an antibody with Cy3-conjugated streptavidin (**A**, top panel). Cellular nuclei were stained with 4’,6-diamidino-2-phenylindole (DAPI) (middle panel). The merged signals indicated that the ERα protein had been translocated into nuclei (bottom panel). These merged fluorescent signals were quantified and statistically analyzed **(B)**. Each value represents the mean ± SEM for *n* = 6. The symbol ^*^ indicates that the value significantly differed from the respective control group, *p* < 0.05.

In untreated human osteoblasts, low levels of ERα were detected (Figure 3A, top-left panel). Nevertheless, exposure of human osteoblasts to estradiol for 1, 6, 12, and 24 h increased amounts of cellular ERα in a time-dependent manner (top panels). Nuclei were detected with propidium iodide dye (middle panels). The merged signals with cyan-blue color indicate colocalization of ERα in nuclei (bottom panels). After administration of estradiol, colocalized signals were time-dependently amplified. Merged signals were quantified and statistically analyzed (Figure 3B). Compared to the control group, treatment of human osteoblasts with estradiol for 12 and 24 h caused 98% and 156% increases in translocation of ERα from the cytoplasm to nuclei.

### Activated ERα induces expressions of specific genomic complex genes

**Figure 4:**
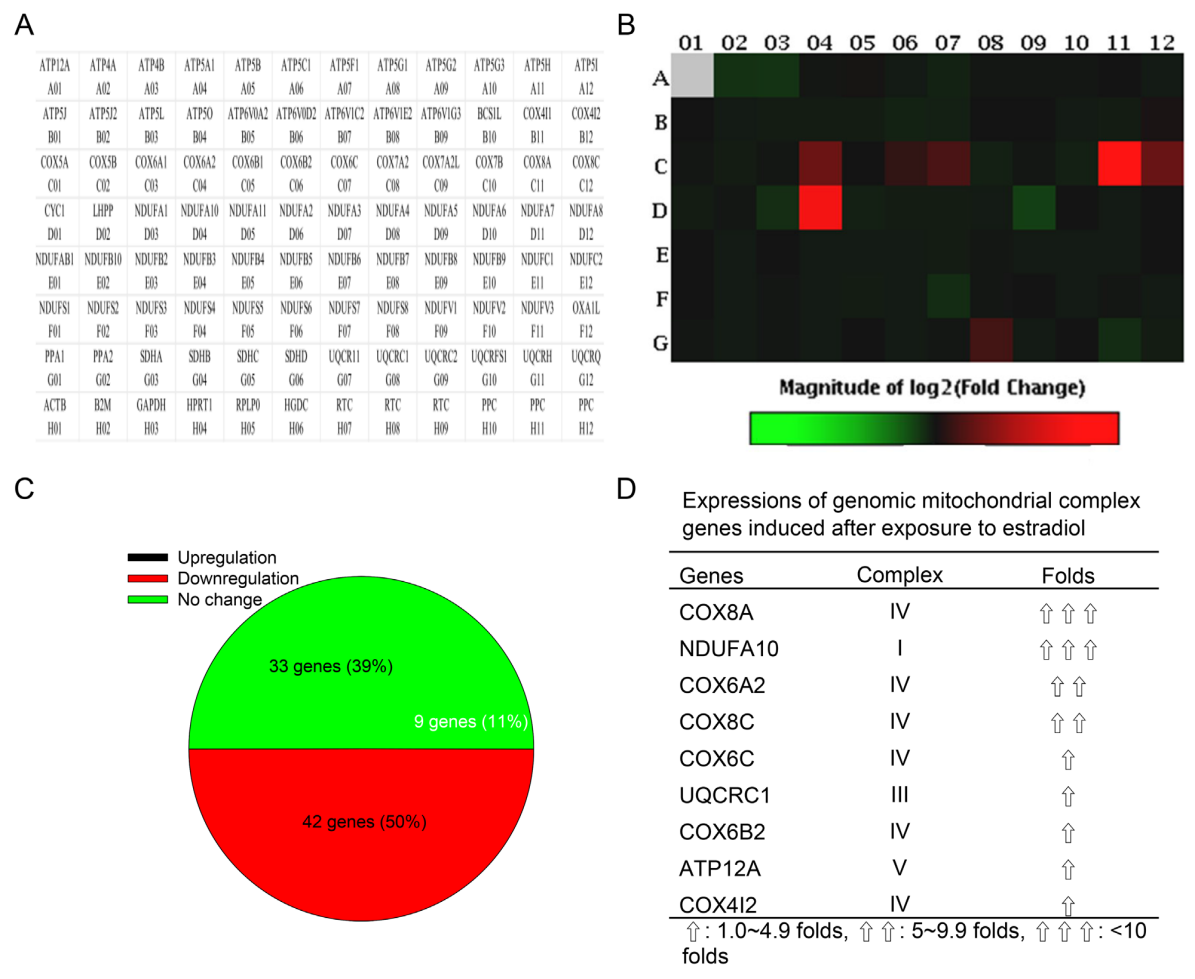
Effects of estradiol on expressions of genomic ATP synthesis-related genes Human osteoblast-like U2OS cells were treated with 10 nM of estradiol for 48 h. Total RNA were isolated for analysis of mitochondrial energy metabolism genes using a PCR array, containing 84 genomic genes encoding certain mitochondrial enzymes for ATP synthesis and 12 loading controls **(A)**. Differential expressions of these genes were measured and shown as a hot map in the order of genes indicated in panel A **(B)**. Percentages of upregulated, downregulated, and unchanged expressions of these genes were further statistically analyzed **(C)**. Also, the major genomic complex genes upregulated by estradiol in human osteoblasts were summarized **(D)**.

In a quantitative PCR array, 87 human mitochondrial ATP synthesis-related genes could be analyzed, including 33 genes for complex I NADH coenzyme Q reductase (*NDUFA1*, *NDUFA10*, *NDUFA11*, *NDUFA2*, *NDUFA3*, *NDUFA4*, *NDUFA5*, *NDUFA6*, *NDUFA7*, *NDUFA8*, *NDUFAB1*, *NDUFB10*, *NDUFB2*, *NDUFB3*, *NDUFB4*, *NDUFB5*, *NDUFB6*, *NDUFB7*, *NDUFB8*, *NDUFB9*, *NDUFC1*, *NDUFC2*, *NDUFS1*, *NDUFS2*, *NDUFS3*, *NDUFS4*, *NDUFS5*, *NDUFS6*, *NDUFS7*, *NDUFS8*, *NDUFV1*, *NDUFV2*, and *NDUFV3*), four genes for complex II succinate coenzyme Q reductase (*SDHA*, *SDHB*, *SDHC*, and *SDHD*), eight genes for complex III coenzyme Q cytochrome c reductase (*BCS1L*, *CYC1*, *UQCR11*, *UQCRC1*, *UQCRC2*, *UQCRFS1*, *UQCRH*, and *UQCRQ*), 14 genes for complex IV cytochrome c oxidase (*COX4I1*, *COX4I2*, *COX5A*, *COX5B*, *COX6A1*, *COX6A2*, *COX6B1*, *COX6B2*, *COX6C*, *COX7A2*, *COX7A2L*, *COX7B*, *COX8A*, and *COX8C*), and 25 genes for complex V ATP synthase (*ATP12A*, *ATP4A*, *ATP4B*, *ATP5A1*, *ATP5B*, *ATP5C1*, *ATP5F1*, *ATP5G1*, *ATP5G2*, *ATP5G3*, *ATP5H*, *ATP5I*, *ATP5J*, *ATP5J2*, *ATP5L*, *ATP5O*, *ATP6V0A2*, *ATP6V0D2*, *ATP6V1C2*, *ATP6V1E2*, *ATP6V1G3*, *LHPP*, *OXA1L*, *PPA1*, and *PPA2*) (Figure 4A). Differential expressions of these human genomic complex genes in human osteoblasts after exposure to estradiol are shown in Figure 4B. Data analyses further showed that estradiol administration induced nine (11%), inhibited 33 (39%), and did not affect 42 (50%) gene expressions in human osteoblasts (Figure 4C). In detail, the upregulated genes were *COX8A* (complex IV), *NDUFA10* (complex I), *COX6A2* (complex IV), *COX8C* (complex IV), *COX6C* (complex IV), *UQCRC1* (complex III), *COX6B2* (complex IV), *ATP12A* (complex V), and *COX412* (complex IV) (Figure 4D).

### Estradiol stimulated translocation of ERα to mitochondria

**Figure 5:**
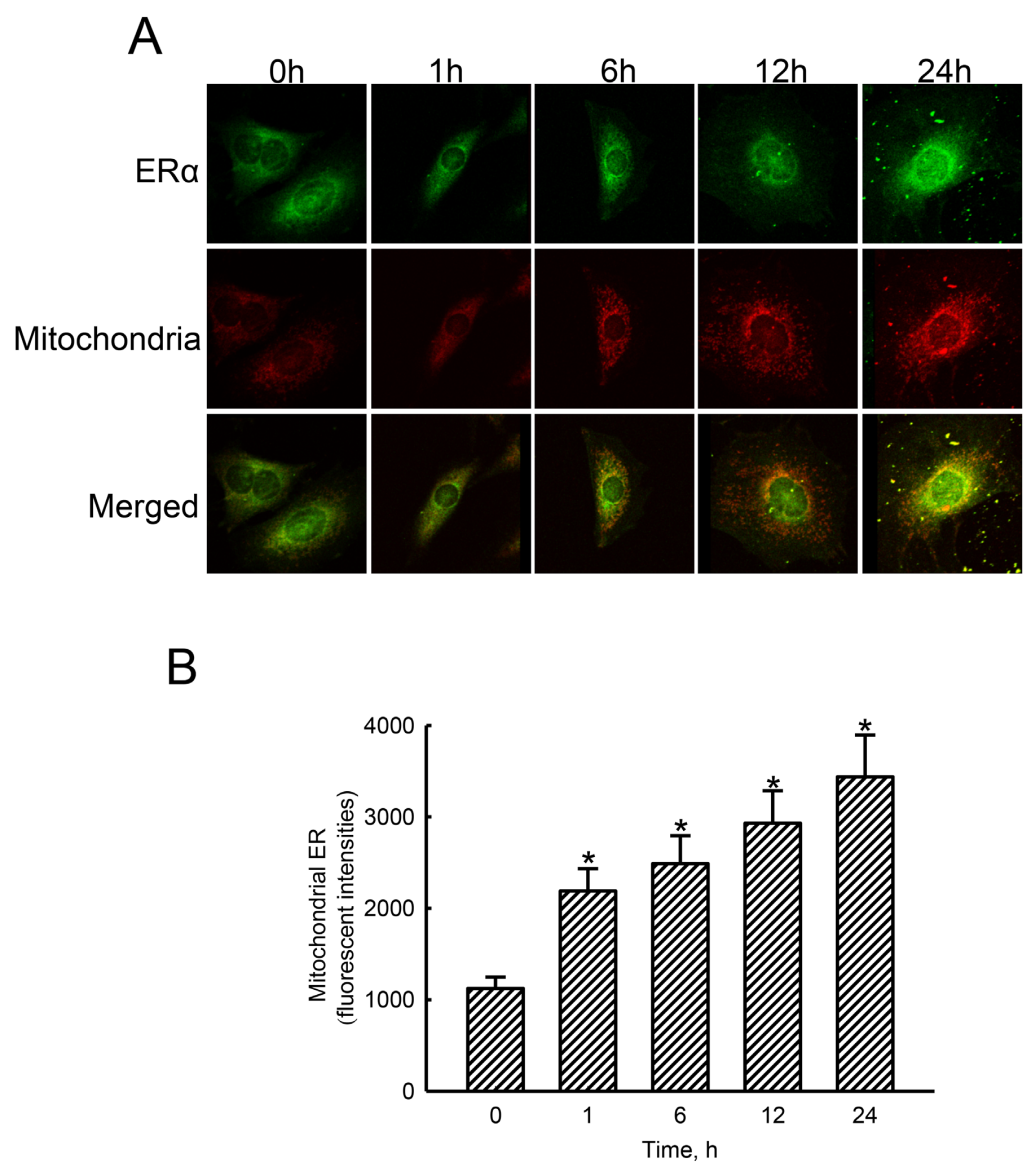
Effects of estradiol on translocation of estrogen receptor alpha (ERα) to mitochondria Human osteoblast-like U2OS cells were exposed to 10 nM of estradiol for 1, 6, 12, and 24 h. Distribution of the ERα protein in human osteoblasts was immunodetected using an antibody with Cy3-conjugated streptavidin (**A**, top panel). Mitochondria of human osteoblasts were stained with 3,3′-dihexyloxacarbocyanine (DiOC6), a positively charged dye (middle panel). Merged signals indicated that the ERα protein had been translocated into mitochondria (bottom panels). These fluorescent signals were quantified and statistically analyzed **(B)**. Each value represents the mean ± SEM for *n* = 6. The symbol ^*^ indicates that the value significantly differed from the respective control group, *p* < 0.05.

Treatment of human osteoblasts with estradiol for 1, 6, 12, and 24 h increased levels of ERα in a time-dependent manner (Figure 5A, top panel). Mitochondria were detected by staining with DiOC6 (middle panels). Merged signals with yellow color indicate colocalization of ERα and mitochondria (bottom panel). These merged signals were quantified and statistically analyzed (Figure 5B). Exposure of human osteoblasts to estradiol for 1, 6, 12, and 24 h led to noteworthy 95%, 122%, 161%, and 206% enhancements in translocation of ERα from the cytoplasm to mitochondria, respectively.

### Participation of ERα in regulating mitochondrial *COX I* and *COX II* gene expressions

**Figure 6:**
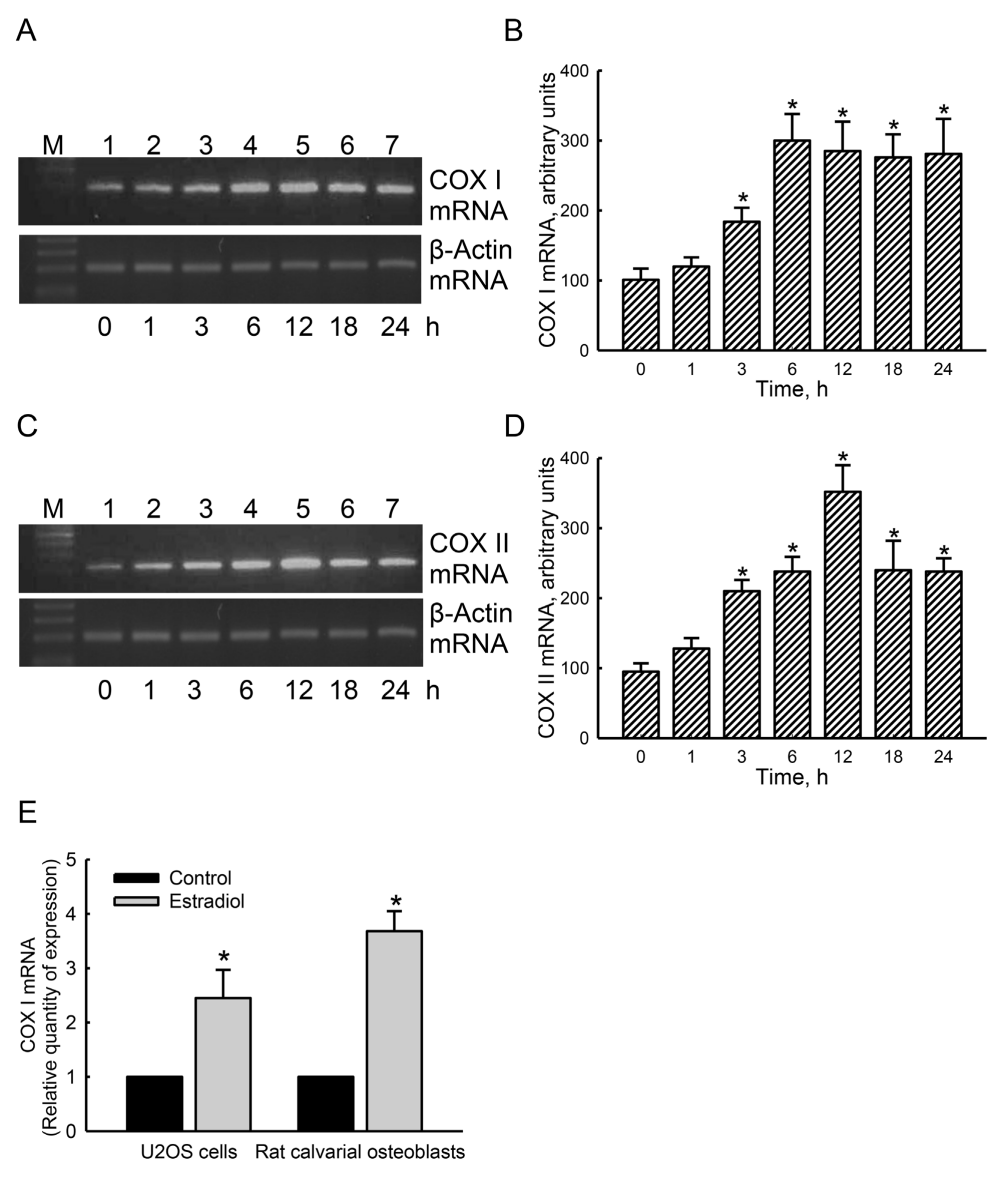
Effects of estradiol on induction of mitochondrial cytochrome c oxidase (COX) I and II mRNA expressions Human osteoblast-like U2OS cells were exposed to 10 nM of estradiol for 1, 3, 6, 12, 18, and 24 h. Levels of COX I and II mRNA were analyzed using an RT-PCR (**A** and **C**, top panels). Amounts of β-actin mRNA were assayed as the internal standard (bottom panel). These bands were quantified and statistically analyzed **(B** and **D)**. A quantitative real-time PCR analysis was carried out to confirm expression of COX I mRNA in U2OS cells and rat calvarial osteoblasts **(E)**. Each value represents the mean ± SEM for *n* = 6. The symbol ^*^ indicates that the value significantly differed from the respective control group, *p* < 0.05.

Our results from a bioinformatics approach revealed that four and six EREs respectively existed in the 5’-promoter regions of the *cox i* and *cox ii* genes. In untreated human osteoblasts, low levels of mitochondrial COX I mRNA were detected (Figure 6A, top panel, lane 1). After exposure to estradiol for 1 h, COX I mRNA was not influenced (lane 2). However, treatment of human osteoblasts with estradiol for 3, 6, 12, 18, and 24 h led to time-dependent induction of mitochondrial COX I mRNA (lanes 3-7). In parallel, exposure of human osteoblasts to estradiol induced mitochondrial COX II mRNA expression in a time-dependent manner (Figure 6C, top panel). β-Actin mRNA was analyzed as the internal controls (Figure 6A, 6C, bottom panels). These bands were quantified and statistically analyzed (Figure 6B, 6D). Treatment of human osteoblasts with estradiol for 3, 6, 12, 18, and 24 h induced mitochondrial COX I mRNA by 1.8-, 3.0-, 2.9-, 2.8-, and 2.8-fold, respectively (Figure 6B). In contrast, amounts of mitochondrial COX II mRNA in human osteoblasts were respectively induced by 2.2-, 2.5-, 3.7-, 2.5-, and 2-fold after exposure to estradiol for 3, 6, 12, 18, and 24 h (Figure 6D). A quantitative real-time PCR analysis was carried out to confirm that estradiol caused 2.5- and 3.7-fold increases in levels of COX I mRNA in human osteoblasts and rat calvarial cells, respectively (Figure 6E).

### Estradiol specifically augmented mitochondrial COX I levels and consequently stimulated mitochondrial bioenergetic activation

**Figure 7:**
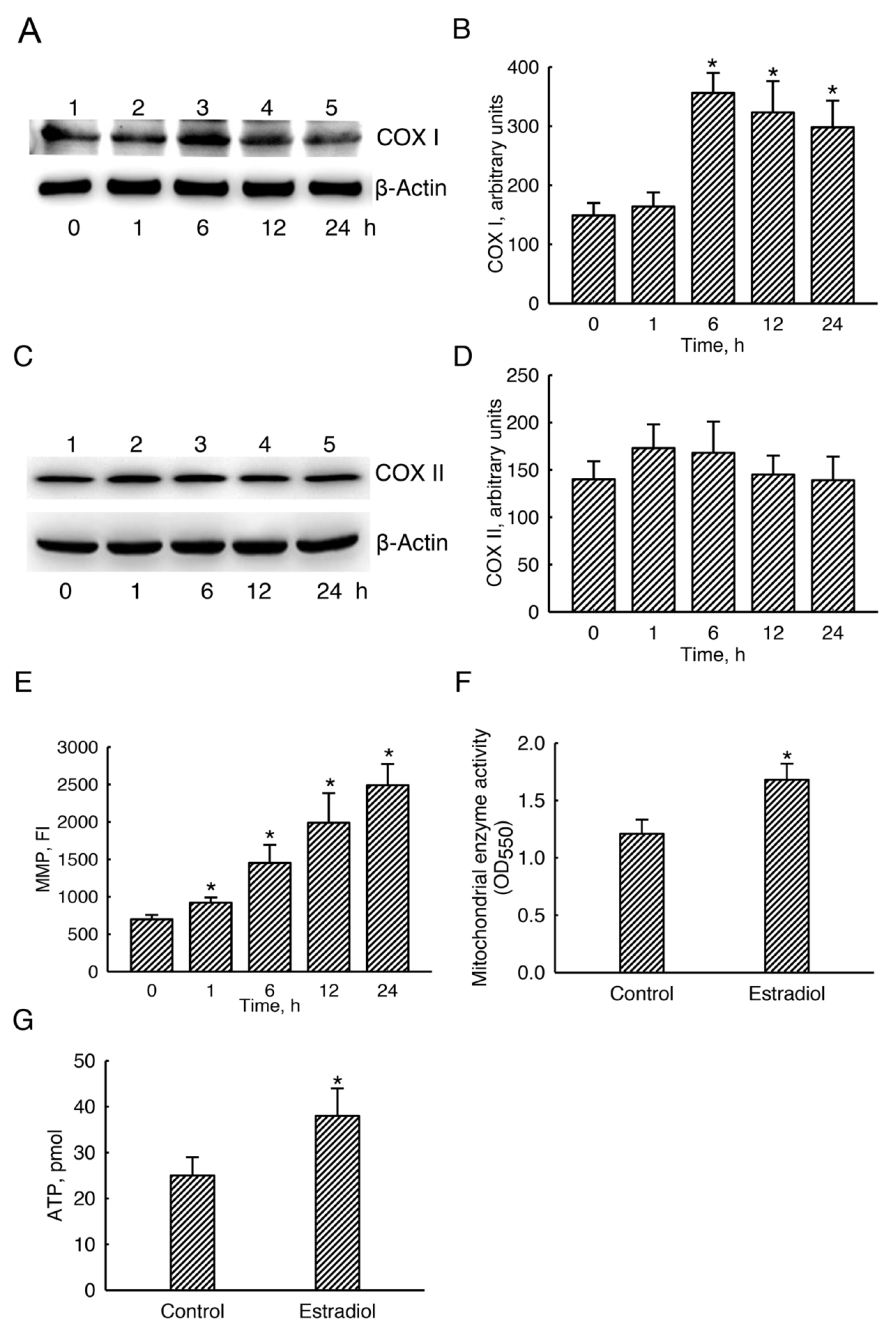
Effects of estradiol on mitochondrial cytochrome c oxidase (COX) I and II protein levels, mitochondrial enzyme activity, and cellular ATP amounts Human osteoblast-like U2OS cells were exposed to 10 nM of estradiol for 1, 6, 12, and 24 h. Levels of COX I and II proteins were immunodetected **(A** and **C, top panels)**. Amounts of β-actin were analyzed as the internal standard **(A** and **C, bottom panels)**. These protein bands were quantified and statistically analyzed (**B** and **D**). Mitochondria of human osteoblasts were stained with 3,3′-dihexyloxacarbocyanine (DiOC6), a positively charged dye. The mitochondrial membrane potential (MMP) of human osteoblasts was determined by quantifying DiOC6-positive signals **(E)**. The mitochondrial enzyme activity was assayed using a colorimetric method **(F)**. Cellular ATP levels were quantified using a bioluminescence assay **(G)**. Each value represents the mean ± SEM for *n* = 6. The symbol ^*^ indicates that the value significantly differed from the respective control group, *p* < 0.05. FI, fluorescent intensity.

Basal levels of mitochondrial COX I were detected (Figure 7A, top panel, lane 1). Exposure of human osteoblasts to estradiol for 1 h did not change COX I levels (lane 2). In comparison, levels of mitochondrial COX I were obviously increased following estradiol treatment for 6, 12, and 24 h (lanes 3-5). β-Actin was immunodetected as the internal standard (bottom panel). These protein bands were quantified and statistically analyzed (Figure 7B). Six, 12, and 24 h later, treatment of human osteoblasts with estradiol led to 2.4-, 2.2-, and 2.1-fold growths in levels of COX I. In contrast, estradiol did not influence mitochondrial COX II levels (Figure 7C, 7D).

Exposure of human osteoblasts to estradiol for 1, 6, 12, and 24 h increased the mitochondrial membrane potential by 32%, 108%, 185%, and 261%, respectively (Figure 7E). In control human osteoblasts, the basal level of mitochondrial enzyme activity was detected (Figure 7F). Nonetheless, treatment with estradiol led to a significant 38% increase in the mitochondrial enzyme activity. Accordingly, compared to the untreated group, exposure to estradiol meaningfully elevated levels of cellular ATP in human osteoblasts by 52% (Figure 7G).

### The estradiol-induced COX I mRNA expression was confirmed with a loss-of-function approach

**Figure 8:**
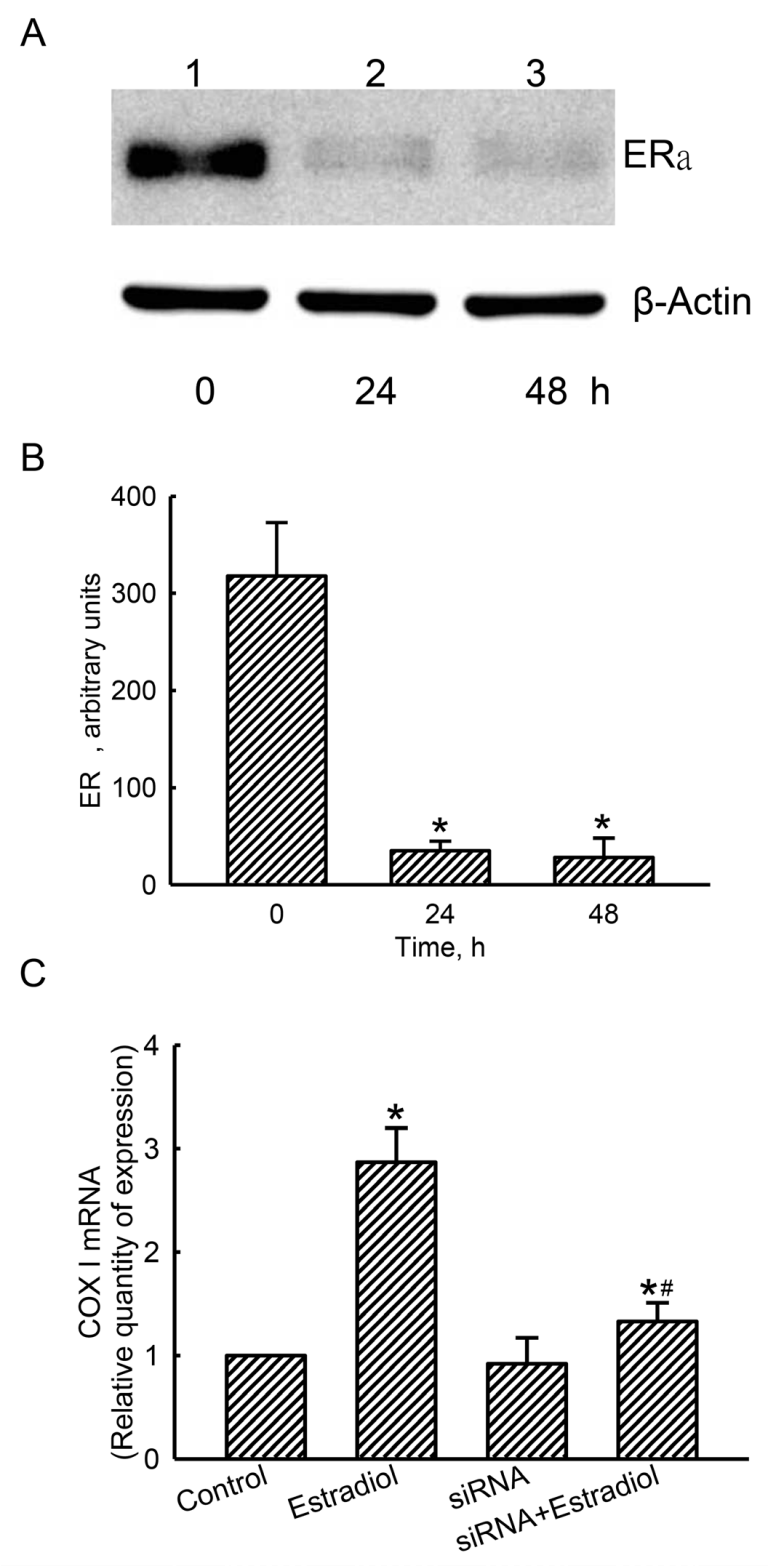
Effects of estrogen receptor alpha (ERα) knockdown on estradiol-induced mitochondrial cytochrome c oxidase (COX) I mRNA expression Human osteoblast-like U2OS cells were treated with ERα siRNA for 24 and 48 h. Scrambled siRNA was administered to control cells as the negative standard. Levels of ERα were immunodetected (**A**, top panel). Amounts of β-actin were analyzed as the internal standard (bottom panel). These protein bands were quantified and statistically analyzed **(B)**. After knocking-down ERα translation for 24 h, human osteoblasts were treated with estradiol for another 6 h. A quantitative PCR analysis was conducted to determine COX I mRNA expression **(C)**. Each value represents the mean ± SEM, *n* = 3. The symbols ^*^ and ^#^ indicate that a value significantly (*p* < 0.05) differed from the control and estradiol-treated groups, respectively.

Compared to the control cells treated with scrambled siRNA, levels of ERα in human osteoblasts were decreased after applying ERα siRNA for 24 and 48 h (Figure 8A, top panel, lanes 1-3). β-Actin was analyzed as the internal control (bottom panel). These protein bands were quantified and statistically analyzed (Figure 8B). Treating with ERα siRNA for 24 and 48 h descend levels of ERα by 89% and 91%, respectively. Exposure of human osteoblasts to estradiol induced mitochondrial COX I mRNA expression by 2.87 folds (Figure 8C). Application of ERα siRNA did not alter basal levels of mitochondrial COX I mRNA in human osteoblasts, but led to a significant 82% inhibition in estradiol-induced COX I mRNA expression (Figure 8C).

## DISCUSSION

This study demonstrated the participation of the estrogen/ERα signaling axis in regulation of osteoblast maturation possibly through stimulating mitochondrial bioenergetic metabolism. Our previous study also reported a similar result that genistein, a phytoestrogen, selectively induced ERα expression in mouse and rat osteoblasts [23]. In parallel with enhancing ERα expression, estradiol led to significant improvements in osteoblast activity, proliferation, and maturation. A continuous process of bone formation, called osteogenesis, comprises three consecutive stages of osteoblast proliferation, differentiation, and mineralization [24, 25]. Hence, estrogen can improve osteoblast mineralization because of its effects of stimulating cell proliferation and cell activity, and this event may take place via an ERα-dependent mechanism. In bone remodeling, maintenance of cellular ATP synthesis is essential for a dynamic balance between osteogenesis and osteoclastogenesis [26]. This study proved that estradiol enlarged the MMP, the mitochondrial enzyme activity, and ATP production in human osteoblasts. Therefore, estrogen can stimulate the mitochondrial bioenergetic system in osteoblasts and thus improve cell maturation. Our preliminary study showed the expression of ERα in human U2OS cells and rat calvarial osteoblasts. Compared to primary osteoblasts, human U2OS cells, derived from a female osteosarcoma patient, were harder to be mineralized. Herein, rat calvarial osteoblasts were used as our model to evaluate the effects of estradiol on ATP synthesis and cell mineralization. Aging can reduce systemic hormone levels and mitochondrial energy metabolism [18]. This study provides an alternative pathway to clarify the roles of the estrogen/ERα axis in aging-induced bone diseases.

Estrogen can improve osteoblast maturation. Osteoblasts, differentiated from stromal stem cells, remarkably contribute to bone formation [4]. Estradiol stimulated proliferation of osteoblasts. Proliferation of osteoblasts and their primitive osteoprogenitors is a vital process in osteogenesis [25]. Hence, estradiol-enhanced osteoblast proliferation is supportive of subsequent cell differentiation and maturation. Moreover, estradiol could elevate ALP activity. ALP, a biomarker of osteoblasts, is regularly assayed to evaluate osteoblast activities [27]. Zhou et al. showed that upregulation in ALP gene expression and enzyme activity is directly correlated with osteoblast differentiation [28]. Corresponding to enhancements in cell proliferation and ALP activity, our present results by Alizarin red S and von Kossa staining protocols further demonstrated that exposure of osteoblasts to estradiol led to significant development of cell calcification. A previous study reported a positive role of ERα in mediating resveratrol-induced proliferation of human bone marrow-derived mesenchymal stem cells and subsequent osteoblastic differentiation [29]. Also, studies from both Heim et al. and our lab stated that the phytoestrogen, genistein, can induce ERα expression and enhance osteogenesis [23, 30]. Herein, expression of ERα was induced in human osteoblasts by estradiol. Thus, estrogen-triggered ERα may partake in maturation of osteoblasts induced by the sex hormone.

Specific genomic complex gene products contribute to estrogen/ERα-induced osteoblast mineralization. In the inner membranes of mitochondria, four complexes and one ATP synthase are anchored together to transfer electrons from the donors of NADH and FADH_2_ via respiratory chain reactions [10, 11]. These complexes are constructed of a variety of complex subunits encoded by chromosomal genes or mitochondrial DNAs [12]. This study showed the translocation of ERα from the cytoplasm to nuclei in human osteoblasts after exposure to estradiol. In nuclei, activated ERα dimers can have strong transcriptional activities to induce expressions of target genes by precisely binding to EREs existing in 5’-promoter regions [14, 21]. Among these induced genomic complex genes, *COX8A*, *COX6A2*, *COX8C*, *COX6C*, *COX6B2*, and *COX412* are subunits for construction of cytochrome c oxidase (complex IV); NDUFA10 is for NADH: ubiquinone oxidoreductase (complex I); and UQCRC1 is for coenzyme Q: cytochrome c oxidoreductase (complex III). Fascinatingly, ATP12A, a subunit of ATP synthase, was also induced by estradiol in human osteoblasts. Bettini and Maggi reported that estrogen can induce cytochrome c oxidase subunit III expression in the rat hippocampus, thus enhancing neuronal activity [31]. This study also demonstrated definite effects of estradiol on ERα expression. Therefore, one of the possible reasons explaining estrogen-induced mitochondrial biogenesis is ERα-involved upregulation of the expressions of precise chromosomal complex I, III, and IV genes.

Mitochondrial COX I contributes to estrogen/ERα-induced energy metabolism. COX I is a mitochondrial-encoded complex enzyme. In complex IV, COX I functions as a core component to build up a redox-active center with other nuclear- and mitochondrial-encoded complex subunits [11]. Estradiol can work as a ligand to bind ERα and stimulate translocation of this activated transcript factor from the cytoplasm to mitochondria in human osteoblasts. In parallel, the *cox i* gene expression were induced by estradiol in both human osteoblasts and rat calvarial osteoblasts. Moreover, a bioinformatic search revealed the existence of EREs in the 5’-promoter region of the *cox i* gene. Concurrently, knocking-down ERα translation using RNAi could inhibit estradiol-induced COX I mRNA expression. Thus, the estrogen/ERα signaling pathway is involved in regulating mitochondrial *cox i* gene expression, and the mechanism may occur at a transcriptional level. Hsieh et al. reported that after trauma-hemorrhage, elevated estrogen levels were associated with increases in ER, COX I, and ATP contents [32]. In breast cancer MCF7 cells, estradiol was shown to increase levels of mitochondrial COX6c and ATP synthase, which may lead to drug resistance of cancer cells [33]. Cytochrome c oxidase is the terminal complex in the mitochondrial electron transport chain, and COX I is a main catalytic subunit in complex IV [34]. Therefore, this study provides cellular and molecular evidence to demonstrate that estrogen/ERα-induced expression of COX 1 participates in regulating mitochondrial bioenergetics and osteoblast mineralization.

Estrogen stimulates mitochondrial activities, thus improving osteoblast calcification. ATP is an end product of aerobic respiratory chain reactions and functionally works as a coenzyme for intracellular energy transfer [10]. The present study demonstrated enhancing effects of estradiol on the MMP and ATP biosynthesis in human osteoblasts. Conservation of the MMP is necessary for respiratory electron transport and subsequent ATP biosynthesis [34]. Hence, estradiol can stimulate ATP production via elevating the MMP. Separately, mitochondrial enzyme activity was instantaneously augmented in human osteoblasts by estradiol. NADH: ubiquinone oxidoreductase, the largest respiratory complex, uses the reducing potential of NADH to drive protons from the matrix across the inner mitochondrial membrane into the intermembrane space to maintain the MMP [35]. Bonora et al. reported that elevation of mitochondrial complex I enzyme activity is closely associated with growth in the respirational rate and ATP synthesis [36]. Consequently, administration of estradiol to human osteoblasts can improve cellular energy production due to successive extensions in complex I enzyme activity, the MMP, and COX I expression. A previous study proved the effects of cellular ATP of stimulating differentiation and maturation of human osteoblast-like Saos-2 cells [37]. Furthermore, a decrease in mitochondrial energy metabolism may be linked to a variety of aging phenotypes, including osteoporosis [38]. The present study implies potential benefits of the estrogen/ERα axis for improving osteoblast maturation and bone formation by upgrading cellular ATP synthesis.

In conclusion, this study has shown considerable benefits of estradiol administration on osteoblast activities, proliferation, and mineralization. Estradiol specifically increased cellular ERα levels and its translocations to nuclei and mitochondria. The nuclear-activated ERα could specifically induce expressions of complex I, III, and IV and ATP synthase subunit genes. More interestingly, estradiol could induce mitochondrial COX I mRNA and protein expressions in osteoblasts via an ERα-dependent mechanism. Consequently, treatment with estradiol increased the MMP, the mitochondrial enzyme activity, and ATP biosynthesis. Taken together, this study showed the participation of the estrogen/ERα axis in osteoblast maturation through improving cellular ATP synthesis because of definite upregulation of expressions of chromosomal and mitochondrial complex subunit genes. However, this study possessed certain research limitations. Our present data cannot explain why estradiol induced COX II mRNA expression but did not affect its protein levels. More experiments, for example, analyzing COX II mRNA-specific RNase polynucleotide phosphorylase activity, are needed in our future study. Furthermore, translational studies using animal models of an ovariectomy and bone defects are being performed in our lab in order to supplementary confirm the roles of the estrogen/ERα signaling alliance in bone formation and fracture healing.

## MATERIALS AND METHODS

### Preparation of rat osteoblasts

Primary osteoblasts were prepared from 3-day-old Wistar rat calvaria following a previously described method [39]. All procedures were performed according to the National Institutes of Health Guidelines for Use of Laboratory Animals and were pre-approved by the Institutional Animal Care and Use Committee of Wan-Fang Hospital, Taipei Medical University (Taipei, Taiwan). Osteoblasts were seeded in Dulbecco’s modified Eagle’s medium (DMEM; Gibco-BRL, Grand Island, NY, USA) supplemented with 10% heat-inactivated fetal bovine serum (FBS), L-glutamine, penicillin (100 IU/ml), and streptomycin (100 μg/ml) in 10-cm tissue culture dishes at 37 °C in a humidified atmosphere of 5% CO_2_. Osteoblasts were grown to confluence prior to drug treatment. Only the first passage of rat osteoblasts was used in this study.

### Cell morphology and ALP activity

Rat calvarial osteoblasts were treated with a differentiation reagent, containing 10 nM dexamethasone, 100 μg/mL ascorbic acid, and 10 mM β-glycerophosphate, and a combination of estradiol and the differentiation reagent for 21 days. β-Estradiol with a purity of >98% was purchased from Sigma (St. Louis, MO, USA). After drug treatment, the cell morphology was observed and photographed using an inverted microscope (Nikon, Tokyo, Japan). The ALP enzyme activity of primary osteoblasts was determined by detecting the formation of p-nitrophenol, a product of p-nitrophenyl phosphate catalyzed by ALP, according to a previously described colorimetric procedure (Sigma) [40].

### Assays of osteoblast mineralization

Osteoblast maturation was determined by evaluating cell mineralization using the von Kossa- and alizarin red S dye-staining protocols as described previously [41]. Rat calvarial osteoblasts were seeded in 6-cm tissue culture dishes and treated with a differentiation reagent, containing 10 nM dexamethasone, 100 μg/mL ascorbic acid, and 10 mM β-glycerophosphate, and a combination of estradiol and the differentiation reagent for 21 days. After drug treatment, the cell morphology was observed and photographed using an inverted microscope (Nikon). Also, osteoblasts were washed with ice-cold phosphate-buffered saline (PBS, 0.14 M NaCl, 2.6 mM KCl, 8 mM Na_2_HPO4, and 1.5mM KH_2_PO_4_) and then fixed in ice-cold 10% formalin for 20 min. For the von Kossa protocol, mineralized matrix was detected by treating fixed cells with 5% silver nitrate for 30 min, followed by subsequent washes with 5% sodium carbonate in 10% formalin for 1 min and 5% sodium thiosulfate for 5 min. The reaction was stopped by washing cells twice with deionized water. For the alizarin red S dye protocol, fixed osteoblasts were thoroughly rinsed and then incubated in 1% alcian blue at pH 2.5 (Thermo Fisher Scientific, Tewksbury, MA, USA) for 12 h. Sections were then incubated in alizarin red S (Thermo Fisher Scientific) for 8 min, dehydrated briefly in xylene, and covered with a coverslip in Permount (Thermo Fisher Scientific). Mineralized nodules were visualized under a light microscope (Nikon). Stained cells were dissolved in dimethyl sulfoxide, and signals were spectrophotometrically measured at a wavelength of 570 nm as described previously [42]. Each experiment was performed in duplicate wells and repeated three times.

### Assay of cell survival

A trypan blue exclusion method was conducted to determine cell survival according to a previous study [43]. Briefly, human osteoblast-like U2OS cells, derived from a female osteosarcoma patient, were purchase from Bioresource Collection and Research Center (Hsinchu, Taiwan). Cells were seeded in 24-well tissue culture plates with DMEM (Gibco-BRL) supplemented with 10% heat-inactivated FBS, L-glutamine, penicillin (100 IU/ml), and streptomycin (100 μg/ml) at 37 °C in a humidified atmosphere of 5% CO_2_. After drug treatment, human osteoblasts were trypsinized with 0.1% trypsin-EDTA (Gibco-BRL). After centrifugation and washing, cells were suspended in PBS and stained with trypan blue dye (Sigma). Fractions of dead cells with a blue signal were visualized and counted using a reverse-phase microscope (Nikon).

### Immunoblot analyses of ERα, ERβ, and β-actin

Immunoblotting protein analyses were conducted following a previous method [44]. After drug treatment, cell lysates from human osteoblast-like cells were prepared in an ice-cold radioimmunoprecipitation assay (RIPA) buffer, containing 25 mM Tris-HCl (pH 7.2), 0.1% sodium dodecylsulfate (SDS), 1% Triton X-100, 1% sodium deoxycholate, 0.15 M NaCl, and 1 mM EDTA. A mixture of 1 mM phenyl methyl sulfonyl fluoride, 1 mM sodium orthovanadate, and 5 μg/ml leupeptin was added to the RIPA buffer in order to avoid degrading the cytosolic proteins by proteinases. Protein concentrations were quantified using a bicinchonic acid protein assay kit (Pierce, Rockford, IL, USA). Proteins (50 μg/well) were subjected to SDS-polyacrylamide gel electrophoresis (PAGE), and transferred to nitrocellulose membranes. Levels of ERα and ERβ proteins were immunodetected using rabbit polyclonal antibodies (pAbs, Santa Cruz Biotechnology, Santa Cruz, CA, USA) against human ERα and ERβ, respectively. Cellular β-actin protein was analyzed using a mouse monoclonal antibody (mAb) against mouse β-actin (Sigma) as the internal control. These protein bands were quantified using a digital imaging system (UVtec, Cambridge, UK) as described previously [45].

### Confocal microscopic analyses of ERα translocation to nuclei and mitochondria

Translocation of ERα in human osteoblast-like cells to nuclei and mitochondria was performed using confocal microscopy according to a previously described method [46]. Briefly, after estradiol treatment, human osteoblasts were fixed with a fixing reagent (acetone: methanol, 1: 1) at -20 °C for 10 min. After rehydration, osteoblasts were reacted with 0.2% Triton X-100 at room temperature for 15 min. A rabbit pAB against human ERα (Santa Cruz Biotechnology) was used in this study to detect ERα in whole cells, including the cytoplasm, nuclei, and mitochondria. Immunodetection of ERα in human osteoblasts was carried out at 4 °C overnight. After washing, cells were sequentially reacted with a secondary antibody and biotin-[define?]SP-conjugated AffiniPure goat anti-rabbit immunoglobulin G (IgG) (Jackson ImmunoResearch, West Grove, PA, USA) at room temperature for 1 h. Following washing, the third antibody with Cy3-conjugated streptavidin (Jackson ImmunoResearch) was added to osteoblasts and reacted at room temperature for 30 min. Nuclei and mitochondria of fixed osteoblasts were respectively stained with 4’,6-diamidino-2-phenylindole (DAPI) and 3,3’-dihexyloxacarbocyanine (DiOC6) (Molecular Probes, Eugene, OR, USA), a positively charged dye, at 37 °C for 30 min. A confocal laser scanning microscope (model FV500, Olympus, Tokyo, Japan) was used for sample observation. The excitation wavelength was set to 568 nm, while a 585-nm long-pass filter was used to collect the emitted light. Illumination of the existence of the ERα protein was demonstrated by the appearance of “hot spots” in the cytoplasm, nuclei, and mitochondria. Images were acquired and quantified using FLUOVIEW software (Olympus). The increased densities of hot spots were analyzed by automated recordings within the same region in a cell. The average density of hot spots was the average of values for 10 areas within a cell.

### Bioinformatic approach

Estrogen response elements (EREs, 13 base pairs, GGTCAnnnTGACC) in the promoter region of the mitochondrial *cox I* and *cox II* genes were predicted using the PROMO bioinformatic system as described previously [46, 47]. Our results respectively indicated that there were four and six predicted ERE sites located in the 5’ promoter regions of the mitochondrial *cox i* and *cox ii* genes.

### Reverse-transcription (RT) and quantitative PCR assays

Expressions of mitochondrial COX I and COX II messenger (m)RNA in human osteoblasts were determined using RT-PCR analyses as described previously [48]. After drug treatment, total RNAs were prepared for analyses of mitochondrial COX I and COX II, and β-actin mRNA. Oligonucleotide primers were designed and synthesized by MDBio (Taipei, Taiwan). Oligonucleotide sequences of the respective upstream and downstream primers were 5’-TACGTTGTAGCCCACTTCCACT-3’ and 5’- GGATAGGCCGAGAAAGTGTTGT -3’ for COX I mRNA; 5’- GTAGTACTCCCGATTGAAGCCC-3’ and 5’-ATTCTAGGACGATGGGCATGAA-3’ for COX II mRNA; and 5’-ATGGATGATGATATCGCCGCGCTCGTCGTC-3’ and 5’-AGGGTGAGGATGCCTCTCTTGCTCTG-3’ for β-actin mRNA [49]. These mRNAs were reverse-transcribed into their cDNAs. Amplification of these COX I, COX II, and β-actin cDNAs was performed with an initial denaturation at 94 °C for 5 min, followed by 35 cycles (94 °C for 45 s, 55 °C for COI and COII and 60 °C for β-actin for 45 s, 72°C for 90 s), a final extension step at 72 °C for 10 min, and a stopping step at 4 °C. PCR products were loaded onto a 1.8% agarose gel containing 0.1 μg/ml ethidium bromide and electrophoretically separated. DNA bands were visualized and photographed under ultraviolet-light exposure. Intensities of the DNA bands in the agarose gel were quantified with the aid of a digital imaging system (UVtec). A quantitative PCR analysis was carried out using iQSYBR Green Supermix (Bio-Rad, Hercules, CA, USA) and the MyiQ Single-Color Real-Time PCR Detection System (Bio-Rad) as described previously [43].

### Protein analyses of mitochondrial COX I and COX II

Protein analyses were carried out according to a previously described method [50]. After drug treatment, cell lysates from human osteoblasts were prepared in ice-cold RIPA buffer. To avoid protein degradation, a proteinase inhibitor mixture, containing 1 mM phenyl methyl sulfonyl fluoride, 1 mM sodium orthovanadate, and 5 μg/ml leupeptin was added to the assay buffer. Protein concentrations were quantified using a bicinchonic acid protein assay kit (Pierce). Proteins (50 μg/well) were subjected to SDS-PAGE, and transferred to nitrocellulose membranes. Amounts of mitochondrial COX I protein were immunodetected using a mouse mAb against human COX I (Abcam, Cambridge, UK). Mitochondrial COX II protein was detected with a rabbit pAb against human COX II (GeneTex, Irvine, CA, USA). Levels of the β-actin protein were immunodetected using a mAb (Sigma) as the internal standard. These protein bands were quantified using a digital imaging system (UVtec).

### ERα knockdown

Expression of ERα in human osteoblasts was knocked-down using an RNA interference (RNAi) method following a small interfering (si)RNA transfection protocol provided by Santa Cruz Biotechnology as described previously [27]. ERα siRNA was a pool of 3 target-specific 20-25-nt siRNAs designed to knock-down ERα’s expression. After culturing human osteoblasts in antibiotic-free DMEM at 37 °C in a humidified atmosphere of 5% CO_2_ for 24 or 48 h, the siRNA duplex solution, which was diluted in the siRNA transfection medium (Santa Cruz Biotechnology), was added to the osteoblasts. Scrambled siRNA, purchased from Santa Cruz Biotechnology, was applied to control cells as a negative standard. After transfection for 24 or 48 h, the medium was replaced with normal DMEM, and the bone cells were treated with estradiol.

### Quantification of the mitochondrial membrane potential (MMP)

The MMP was determined following a previously described method [51]. Briefly, human osteoblasts (5 x 10^5^ cells) were seeded in 12-well tissue culture plates overnight. After administration of estradiol, osteoblasts were harvested and incubated with 3,30-dihexyloxacarbocyanine iodide (DiOC6), an intracellular green-fluorescent probe specifically used to detect the MMP in live cells, at 37 °C for 30 min in a humidified atmosphere of 5% CO2. After washing and centrifugation, cell pellets were suspended in PBS. Intracellular fluorescent intensities were analyzed using a flow cytometer (Olympus).

### Assay of the mitochondrial enzyme activity

The mitochondrial enzyme activation was assayed by determining activity of NAD(P)H-dependent cellular oxidoreductase enzymes using a colorimetric method [23]. After administration of estradiol and the differentiation agent, human osteoblasts were cultured with a new medium containing 0.5 mg/ml 3-(4,5-dimethylthiazol-2-yl)-2,5-diphenyltetrazolium bromide for a further 3 h. Blue formazan products in the osteoblasts were dissolved in dimethyl sulphoxide and spectrophotometrically measured at a wavelength of 550 nm.

### Measurement of cellular ATP

The levels of cellular ATP in human osteoblasts were determined by a bioluminescence assay based on the luciferase requirement for ATP in producing emission light, according to the protocol for the Molecular Probes ATP Determination Kit (Molecular Probes, Eugene, OR) as describe previously [52]. The luminant light (560 nm) emitted by the luciferasemediated reaction of ATP and luciferin was detected by a WALLAC VICTOR® 1420 multilabel counter (Welch Allyn, Turku, Finland).

### Statistical analysis

Statistical differences between the control and drug-treated groups were considered significant when the *p* value of Duncan’s multiple-range test was < 0.05. Statistical analyses between drug-treated groups were carried out using two-way analysis of variance (ANOVA).
